# Gold Foil

**Published:** 1869-11

**Authors:** G. V. Black


### ARTICLE VI.

### *Gold Foil.*

By Dr. G. V. Black.

The first important question to the dentist after having received his gold foil from the beater or dealer is, how can it best be kept in good working order. If it is not kept in good condition, it matters little how or in what form it may be prepared, all operations with it will be faulty.

First, then, in learning how best to preserve the working properties of the gold, it becomes important to study intimately those influences which may be injurious, learn, as far as possible, what they are, how they act, and where they come from; then we may find the proper mode of counteracting them. First, we find that gold possessed of good welding properties gradually loses that important requisite by continued exposure to the ordinary atmosphere. In some cases this may be due to dust collecting upon it in such a manner as to prevent the surfaces from coming into intimate contact, thus preventing a union; but we have abundant proof that there is a far more subtle influence than this, and one far more difficult to counteract, for dust may be excluded with comparative ease.

The fact that gold loses its welding property by exposure to the atmosphere, points us directly to the effects of the gases upon it, especially those that may be found in the air as impurities; for the reason that by close observation we find that the asmospheric effect differs very materially at different times. You all know how common it is to find your gold, which worked well yesterday, work very badly to-day. Why this difference? is a very important question. It will be my effort to point out some of the causes for these differences, and the remedies, as far as I may be able. In prosecuting this enquiry, I have instituted a long series of experiments, in which I find certain gases neutral, others decidedly injurious, while others again seem possessed of a beneficial action.

Among the neutral gases stand all the simple or elementary gases except chlorine, the action of which is remarkable, and will be briefly considered farther on. Among those detrimental, all those gases containing phosphorus or sulphur stand prominent, while ammoniacal gas seems to stand almost alone as beneficial.

Many substances are neutral which might be supposed to be injurious. For instance, gold foil soaked in tincture of iodine, dried, and then annealed, is rather improved than injured, if any difference. The same is true of carbolic acid, ether, chloroform, alcohol, water, and most other substances usually found about the the dentist's case.

The neutral gases need not be considered, but I will notice a few of those decided actions, giving the form of an experiment with each.

Sulphurous acid gas is produced directly from the burning of sulphur in the air, and is composed of one atom of sulphur to two of oxygen; is possessed of decided acid properties, and has the smell of a burning match, and is produced in the dentist's office every time a match is lighted.

*Experiment.*—A test tube filled with this gas by heating together oxide of manganese and flowers of sulphur, and some ropes of gold foil, part annealed and part soft, introduced. After standing twenty-four hours, it was removed and examined. The soft gold, on being annealed, gave off the gas sufficiently to be very noticeable to the smell, and with a slight crackling sound. The welding property was tried, and although it adhered somewhat, it was easily pulled apart again. The annealed gold was found perfectly soft—would not weld any more than so much tissue paper. On being re-annealed, it gave off the sulphurous fumes, &c., as the soft gold, but there was no restoration of welding property by means of heat. You will notice the difference in the action of this gas on annealed and unannealed gold; and I will state that this distinction was plainly marked in all my experiments with this gas and some of the others.

And when I come to speak more particularly of annealing, I will give some reasons why it should be so.

This gas is a dangerous one to the operator, for it is not only produced from burning matches, but is constantly exhaled from all sorts of rubber goods, with which the dentist's office is generally well stocked now-a-days. It is given off in very appreciable quantities in the trimming and finishing of rubber plates, in vulcanizing, and in all the operations with rubber, and wherever it may be. The truth of these statements is easily tested. For this purpose I placed some litimus paper in a drawer containing the rubber used as coffer dam; in a few days it was completely reddened. Some gold placed in the same drawer was affected similar to that exposed to pure sulphurous acid, though not to so great an extent. In many of our cities the air is often rendered more or less sulphurous by the burning of sulphurous coal, and from other causes the air is often charged with this gas.

It is a somewhat curious fact that sulphur may be wrapped in a rope of gold foil and then burned out without injury to the gold, but this becomes plain when we find that this gas will condense on gold, or affect it only at comparative low temperatures.

*Phosphorus experiment.*—A bottle was filled with phosphoric acid by burning phosphorus in oxygen gas, and some ropes of gold foil hung within for a few hours, after which it was examined. The annealed gold was found soft, and re-annealing failed to re-develop the welding property. The unannealed gold also refused to weld after having been annealed, even at a bright red heat. After the bottle had remained uncorked ten or twelve hours, more gold was hung in it with precisely the same result. After the bottle had stood for one week, more gold was hung in it, and the effect was the same. Phosphous is employed in making matches, and is given off in the form of this gas whenever they are burned; it is also formed from the spontaneous combustion of phosphoretted hydrogen gas, which is produced from the decay of animal and vegetable matter, together with sulphuretted hydrogen. Thus we see we cannot consider ourselves entirely free from it at any time, and that it will ruin the welding property of our gold.

*Sulphuretted hydrogen experimint.*—A test tube was filled with sulphuretted hydrogen by decomposing muriatic acid with sulphuret of antimony, and some ropes of gold, part annealed and part soft placed within. After twenty-four hours the gold was removed and examined. No change in the appearance of the gold was observed. On bringing the rope into a strong alcoholic flame there was a distinc crackling sound, and the rope was burst open in a number of places by the escaping gas, which burned visibly. It does not seem to be driven off until a bright red heat is produced. The welding property could, in no one of these experiments, be reproduced by re-annealing the pre-annealed gold. In some cases there was a feeble show of adhesiveness in the unannealed gold, after annealing, but not enough to be of any practical advantage. After digesting the rope in aqua ammonia for a few minutes, and then drying at a gentle heat, a white sublimate is obtained on annealing, which may be collected on a cold glass held above. This is probably the sulphide of ammonia. If any one should be skeptical in regard to the actual condensation of the gases upon gold foil, this experiment must be sufficient proof to do away with all doubts, particularly when a similar phenomenon occurs with some other gases. This gas is exhaled in large quantities from all decaying animal substances, and the air generally contains some of it, though the proportion is usually small. But it is clear that it has a tendency to condense upon the gold, and may be collected upon it from the air. And then it may be actually produced in the dentist's office, Blood left in a spittoon over Sunday, or even over night, in warm weather, may exhale enough of this gas to spoil all the gold exposed to its action. It may be exhaled by old teeth, or any animal matter whatever, which may, by any oversight, be left until putrefaction takes place.

The effect produced by burning matches seems to be very irregular, sometimes being that of sulphurous and again that of phosphoric acid, and sometimes different from either. The effect is almost uniformly ruinous on recently annealed gold ; but soft gold will sometime weld after being subjected to it and then annealed, and again it refuses to do so, and again it occasionally happens, that amonia fails to effect a restoration. The ruinous effects of the exhalations from the skin are well known to the profession, and need not be dwelt upon in this paper. My experiments confirm the generally received idea that they are always injurious, and further, that if the gold be long exposed to them, the welding property will be entirely destroyed.

Many other gases might be mentioned, but I have discovered no others which are liable to be brought in contact with gold, that exert any decided injurious effect.

Carbolic acid gas softens annealed gold, but the welding property is immediately restored by heat.

Common illuminating gas produces a peculiar spotting of the gold, making it look as if mercury had been sprinkled over it ; these spots disappear before a moderate heat, and the gold is apparently uninjured.

Ammonical gas, or its solution in water, is found to be rather beneficial than otherwise, in its action on gold foil. It condenses on and removes the welding property of cohesive gold ; but a very moderate heat will remove the ammonia, and cause the cohesiveness to return. When this property is lost by contact with some one of the gases mentioned, the condition may be improved, and the welding property entirely restored, in many cases, by treating it with this gas. It will be seen that all those gases which are liable to come into contact with our gold and injure it are acid gases, which combine readily with ammonia as an alkaline base to form certain salts. Those containing sulphur form sulphides or sulpates of ammonia, while phosphoric acid forms phosphate of ammonia.

With these facts before us, we cannot be at a loss for a moment in regard to its use. If ammonia be present in excess, we may be sure that any of the gases mentioned will be neutralized, and our gold perfectly protected.

It thus becomes an ever wakeful watch-dog, ready to step in at our bidding, take possession of our property, and destroy any intruder before harm can be done, and again deliver it up to us when it may be wanted.

A small bottle of ammonia set in the gold drawer, loosely corked, will be found sufficient to protect our gold from all harm.

*Chlorine.*—Chlorine gas, from its very decided action, possesses a peculiar interest, although it is not met with in the free state, and, therefore, is not liable to come in contact with our gold. I will give merely a synopsis of its peculiarities.

A bottle was filled with chlorine gas by the usual method and a number of sheets of gold, in the form of rope, placed within it. After a few hours they were examined. The lustre of the gold was very perceptibly changed, being dull and of a different shade of yellow; when brought in contact with the skin a deep stain was produced, resembling that of iodine. The instrument with which it is handled turns black. Litmus paper is reddened quickly when both are dry ; damp litmus, bottled with it in a clear bottle, is first reddened, then turns almost white. Paper dampened with a solution of iodide of potassium and starch is first blackened, but afterward shows a variety of colors, among which a very brilliant yellow is conspicuous; a piece of black velvet was changed to a very light brown; a small piece of the same was put into a very small tube, and the gold pressed in against it—after a few hours it was almost a clear white; the same was repeated with the gold and cloth perfectly dry—several days were required to produce the same result. The welding property of the gold was entirely lost, but was completely restored by re-annealing; when heated to redness a copious green flame is given off, which turns red when the heat is intense, presenting a beautiful appearance. When soaked in aqua ammonia, slowly dried, and then annealed, a white sublimate is obtained, which may be collected upon a cold glass held above—a single sheet of gold giving off enough to form a complete crust over a space of two to three inches in circumference; this substance is probably the chlorohydrate of ammonia. When a little water is poured over the gold in a test tube, it takes a yellowish color, almost identical with that of olive oil. I find that on the addition of water, the chlorine instantly attacks the gold and dissolves a portion of it, which it carries into the water, giving rise to this peculiar color; pure chlorine water is almost perfectly clear. Gold subjected to any of the sulphur gases is cleaned, and the welding property restored by subjecting it to chlorine and then annealing; but it fails in case of phosphoric acid.

I had hoped, in the first of my experiments with this gas, that gold treated with it might be employed for the purpose of bleaching discolored teeth; but the fact of its carrying a portion of the gold with it when moisture is present precludes its use, on account of the brownish stain imparted by the gold. I hope this difficulty may be overcome, thus giving us a safe and efficient bleaching agent, which may be used without the least inconvenience to the patient.

*Annealing.*—The annealing of gold has received considerable attention from the profession, but perhaps not quite as much as it deserves, considering its great importance. The cdanges effected in gold foil by heat have been differently explained by different persons, and as yet there seems to be no received or fully accepted theory in regard to it. It is true we all know that the direct effect of annealing is to develop the welding property, and if this fails we conclnde that there is something wrong with the gold; it has been thought that heat acted upon the arrangement of the atoms of the metal, producing such a condition that when brought into contact with a similar arrangement, the particles composing the two surfaces would interlock, and be held firmly together. This theory might seem to be supported by the fact that heat applied to large pieces of gold, or to plate, has the effect to fasten them, evidently producing some molecular change—yet that there is another cause for the development of this peculiar property, I think is fully proven by the effects of the gases.

*The welding of pure gold foil is prevented by the gases being condensed on its surface, thereby preventing intimate contact; the direct effect of annealing is to drive off such gases, and render the surfaces clean.* To prove this, take a rope of gold foil, anneal it in a bath of dry carbonic acid gas for an hour or more; upon trial its cohesiveness is gone; you may put as much force upon it as you like, it will not weld, but is, perhaps, much softer than before annealing; bring this piece again under the influence of the ordinary annealing heat, and the property returns at once and as perfectly as before.

This experiment may be varied by substituting other gases for the carbonic acid, with a like result; some of the gases, however, refuse to be entirely driven off by the ordinary annealing heat, and if we experiment with them, the welding property does not return, as we have seen in the foregoing.

In case of chlorine, the condensation is so great as to perceptibly change the color of the metal, and when expelled by heat the voluminous green flame gives us the idea of a very considerable condensation. While the gas is on the gold, it is perfectly soft—will not stick together any more than tissue paper, though it might have been in a fine cohesive state only a few minutes before; as soon as the gas is driven off the property returns.

There seems to be a question as to the best heat for annealing. This, in my opinion, will depend entirely on the gas that may have possession of its surface. If it be carbonic acid—which is usually the case—the heat need not be raised quite to redness, for we find that gas to be driven off at a low temperature; if it be sulphuric acid, it should be heated as hot as possible without melting the gold, and kept so for some minutes, and even then it may not be perfectly cleaned. This is very much like the melting point of metals, or the boiling point of liquids—some melt at one point, some at another; some liquids boil at one point, and some at another. It is the same with the gases on gold, some are drawn off at a low temperature, while with some others the metal must actually melt before they leave it.

The gases do not leave the gold instantly at a given temperature, but the first is expelled at a comparatively low heat, and they continue to leave it as the heat rises, until they are completely driven off. This enables us to acquire any degree of adhesiveness we may desire. Some parts of a filling may best be made of non-adhesive gold, while other parts require a perfectly coherent mass. By practice all the gradations of adhesiveness may be attained and used with perfect facility; for this reason the alcohol flame and the annealing of each piece of gold as it may be wanted for use is to be preferred over all other methods.

It has become a custom to say that annealing gold hardens it. This is only true in appearance; the gold is actually softened, just as it is in plate. The gold appears stiffer for the reason that the laminæ of foil stick together whenever they come in contact, instead of sliding easily upon each other as in the case of unannealed gold; this gives the impression of increased hardness, while the facts are just the reverse.

The use of gold rendered adhesive by the beater seems to me to be unadvisable, for the reason that such gold is affected more and easier by deleterious agents in the air than soft gold. In all my experiments there has been a marked difference in intensity and quickness of action of the gases on annealed and unannealed gold ; the recently annealed gold always suffering most. This is explained by the fact that any deleterious gases coming in contact with it finds its surface and its pores, (if they exist,) unoccupied , and consequently no resistance to its action. All plastic golds are affected by the gases to a far greater extent than foils, for the reason that their structure is so much better calculated to absorb and retain them. This needs no further explanation, being palpable to every one ; it makes the great difficulty of keeping these forms of gold in good working condition quite clear. I think that if those preparing this form would keep it strongly scented with ammonia, and the dentist do the same, until it is ready to be annealed preparatory to using, this difficulty would be entirely overcome.—*Missouri Dental Journal.* *To be Continued.*

---

